# Biophysical Mode-of-Action and Selectivity Analysis of Allosteric Inhibitors of Hepatitis C Virus (HCV) Polymerase

**DOI:** 10.3390/v9060151

**Published:** 2017-06-16

**Authors:** Eldar Abdurakhmanov, Sara Øie Solbak, U. Helena Danielson

**Affiliations:** Department of Chemistry—BMC, Uppsala University, SE-751 23 Uppsala, Sweden; eldar.abdurakhmanov@kemi.uu.se (E.A.); sara.solbak@kemi.uu.se (S.Ø.S.)

**Keywords:** hepatitis C virus (HCV), non-structural protein (NS) polymerase, genotypes, allosteric inhibitor, surface plasmon resonance (SPR)

## Abstract

Allosteric inhibitors of hepatitis C virus (HCV) non-structural protein 5B (NS5B) polymerase are effective for treatment of genotype 1, although their mode of action and potential to inhibit other isolates and genotypes are not well established. We have used biophysical techniques and a novel biosensor-based real-time polymerase assay to investigate the mode-of-action and selectivity of four inhibitors against enzyme from genotypes 1b (BK and Con1) and 3a. Two thumb inhibitors (lomibuvir and filibuvir) interacted with all three NS5B variants, although the affinities for the 3a enzyme were low. Of the two tested palm inhibitors (dasabuvir and nesbuvir), only dasabuvir interacted with the 1b variant, and nesbuvir interacted with NS5B 3a. Lomibuvir, filibuvir and dasabuvir stabilized the structure of the two 1b variants, but not the 3a enzyme. The thumb compounds interfered with the interaction between the enzyme and RNA and blocked the transition from initiation to elongation. The two allosteric inhibitor types have different inhibition mechanisms. Sequence and structure analysis revealed differences in the binding sites for 1b and 3a variants, explaining the poor effect against genotype 3a NS5B. The indirect mode-of-action needs to be considered when designing allosteric compounds. The current approach provides an efficient strategy for identifying and optimizing allosteric inhibitors targeting HCV genotype 3a.

## 1. Introduction

About 3% of the human population worldwide is infected with hepatitis C virus (HCV). Approximately 70% of these individuals develop a chronic HCV infection, one of the main causes of liver cirrhosis and hepatic cancers [1]. The virus exhibits a high genetic diversity, with six major genotypes and many subtypes identified on the basis of phylogenetic and sequence analyses of whole viral genomes. They have different geographic distributions [2], with genotype 1 being the most widespread and predominant genotype in most regions. However, genotype 3 is the more predominant genotype in Asia, with a very high prevalence in Pakistan [3,4] and also in Scandinavia, where about 50% of HCV-infected individuals have genotype 3. This genotype is not only quite widespread geographically, but it is also associated with a high rate of liver fibrosis progression and liver cancer development [5,6]. Genotype 3 infections are currently the most difficult to treat, but clinical trials have shown that they can be effectively treated with a combination of the nucleoside analogue sofosbuvir and the non-structural protein 5A (NS5A) inhibitor velpatasvir (Epclusa) [7]. However, this therapy is expensive and the occurence of resistance-associated variants remains a challenge.

HCV treatment was until recently limited to indirect and general therapy based on PEGylated interferon-α and ribavirin. It is an expensive and poorly tolerated therapy with varying degrees of sustained virological response for different genotypes, however this therapy is fairly effective against genotype 3 infections. The newer direct acting antivirals (DAA) targeting the viral protease or polymerase have many advantages, but are not equally effective in all patient groups [8]. For example, simeprevir (Olysio, TMC435) is a second-generation NS3 protease inhibitor with antiviral activity against genotypes 1, 2, 4, 5 and 6, but not genotype 3 [9]. Sofosbuvir (Sovaldi, GS-7977), a nucleotide inhibitor of the NS5B polymerase, has become standard of care for the majority of patients with HCV infections as it has a good effect on all genotypes, a high barrier to resistance and favorable clinical pharmacology profile. Although sofosbuvir is considered to have pan-genotypic inhibitory activities, its treatment efficacy against HCV genotype 3 is lower than against the other genotypes [10]. In particular, its efficacy in genotype 3 patients with cirrhosis is not satisfactory. Dasabuvir (Exviera, ABT-333), a non-nucleoside inhibitor of the NS5B polymerase developed for treatment of genotype 1 infection, was recently approved by the Food and Drug Administration (FDA) [11,12]. It is only administered together with the NS5A inhibitor ombitasvir, and the HCV protease inhibitor paritaprevir is given with low-dose ritonavir for a pharmacologic boosting effect. Despite the many advantages of the new DAAs, some compounds require co-administration with other drugs, and, in addition, they lack effectiveness against rapid emergence of drug resistant variants [13,14]. The search for next-generation DAAs thus needs to be continued [15]. To serve as effective complements to current drugs, it is perceived that they should have novel modes of action, potentially avoiding current side effects and resistance problems, and ideally being more cost-effective in the clinic.

The development of DAAs has focused on viral polymerases, as they play a fundamental role in replication. The RNA-dependent RNA-polymerase (RdRp) of HCV is an endoplasmic reticulum membrane-associated protein, with a membrane spanning a C-terminal helix consisting of 21 amino acid residues. The enzyme shares a common “right hand” structure with other RdRps and has three main domains: fingers, palm and thumb. However, the structure is unusual since it has a narrow active site as a result of a close interaction between the fingers and thumb domains [16,17]. NS5B functions in an oligomeric form and catalyzes the synthesis of both positive and negative RNAs during viral replication [18]. The enzyme is an error-prone RdRp, lacking proof-reading activity, which explains the large number of genomic subtypes and rapid evolution of resistant variants [19].

**Figure 1:**
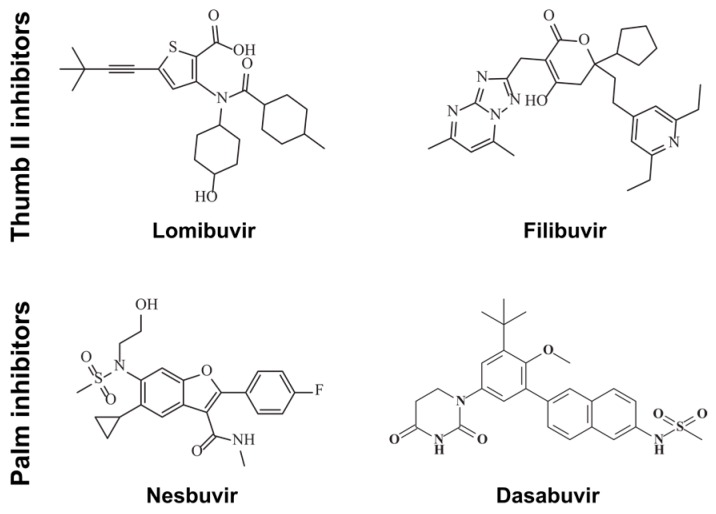
Structures of studied non-nucleoside thumb II and palm inhibitors.

In order to avoid problems associated with nucleotide analogues such as inhibition of other essential human enzymes, for example, mitochondrial DNA polymerases, RNA polymerases, kinases, DNA methyltransferases, and some others [20,21], polymerase inhibitors that target allosteric sites have become popular. Five distinct allosteric binding pockets have been identified in NS5B, two in the thumb domain, two in the palm domain and one in the finger domain. Two allosteric non-nucleoside thumb II site inhibitors, filibuvir (PF-00868554) and lomibuvir (VX-222) (Figure 1) have shown good potencies, but their development has been paused for various reasons [22]. However, recently TREK therapeutics announced a further development of lomibuvir [23]. Also, allosteric palm site inhibitors of NS5B have been developed (Figure 1). Nesbuvir (HCV-796) was an early compound now discontinued due to liver toxicity. However, it indicated the possibility to develop compounds with pan-genotypic activity [13]. Dasabuvir is a more recent compound, but the FDA has only approved it for use in combination with other anti-HCV DAAs and ritonavir, originally an anti-human immunodeficiency virus (HIV) protease inhibitor [24].

Due to the indirect mode-of-action of allosteric inhibitors, the molecular details of binding and inhibition are often elusive. Recently, Deredge et al. proposed a detailed mechanism of action of thumb II inhibitors, however, palm inhibitors and other genotypes were not compared in this study [25]. Although the binding sites for filibuvir and nesbuvir are known from crystal structures of the inhibitors in complex with NS5B [26,27], they are not available for lomibuvir and dasabuvir [13]. A disadvantage of allosteric inhibitors is that they often bind to sites that are not critical for the function of the enzyme and inhibition can therefore be circumvented by single mutations that do not influence catalysis. Resistance substitutions in thumb II and palm allosteric sites that have an effect on RNA synthesis in the presence of thumb II and palm inhibitors have been identified [13,28,29]. Similarly, non-critical allosteric sites are not conserved between genotypes, which is demonstrated by the fact that most non-nucleoside NS5B inhibitors are not effective against genotype 3 HCV [30].

In this study, we sought to investigate a set of allosteric inhibitors (Figure 1) and compare their interaction characteristics and effects on thermal stability and RNA binding, using NS5B polymerase from genotypes 1b (isolates Con1 and BK) and 3a. We further developed assays previously established for the discovery of allosteric inhibitors targeting genotype 1b NS5B [31] now for the discovery of allosteric inhibitors targeting also NS5B genotype 3. Furthermore, we for the first time describe a real-time NS5B polymerase assay performed on a surface plasmon resonance biosensor (SPR) chip. Our results enabled the interpretation of the efficacies of the allosteric inhibitors against the enzymes from different genotypes. Sequence analysis and structural predictions further explain why the compounds are not effective against NS5B 3a, which may be used to guide development of efficient drugs against HCV genotype 3a infections.

## 2. Materials and Methods

### 2.1. Inhibitors

Filibuvir (PF-00868554) was synthesized according to the procedure described by Li et al. [26] and lomibuvir (VX-222) according to the international patent application WO2008/058393. Nesbuvir (HCV-796) was obtained from Gerhard Pürstinger (University of Innsbruck, Innsbruck, Austria) via the European Vigilance Network for the Management of Antiviral Drug Resistance (VIRGIL) Dasabuvir (ABT-333) was purchased from MedChemExpress. All compounds had >95% purity as assessed by HPLC.

### 2.2. Protein Production

The ectodomain of NS5B (NS5B Δ21) was produced as earlier described, with the details of collection of blood samples, viral RNA extraction, cDNA formation, and cloning of NS5B 3a DNA into an expression vector published by Ehrenberg et al. [32] and methods for protein expression and purification published by Winquist et al. [31].

### 2.3. Differential Scanning Fluorimetry

The effect of the allosteric compounds on the thermal stability of NS5B was assessed by differential scanning fluorimetry (DSF), performed as described previously by Winquist et al. [31]. In brief, NS5B Δ21 was mixed with SYPRO Orange (Invitrogen, Carlsbad, CA, USA) and inhibitors in a total volume of 40 µL in transparent 46 well plates. Each sample contained 2 µM of protein and 10 µM of inhibitor, with a final concentration of dimethyl sulfoxide (DMSO) up to 2% in a 50 mM Tris-HCl buffer, pH 7.5. The temperature was increased by 0.5 °C per 1 min from 22 °C to 85 °C. Experiments were performed in triplicates using a MiniOpticon real-time PCR instrument (Bio Rad Laboratories). The fluorescence was monitored using excitation at 494 nm and emission at 521 nm. The thermal melting points of the enzymes (*T*_m_) were calculated using Bio-Rad CFX Manager software, v. 1.5 (Bio Rad Laboratories, Hercules, CA, USA).

### 2.4. Biosensor Analysis of Inhibitor–NS5B Interactions

The interaction between the inhibitors and NS5B was analyzed using Biacore S51 and T200 instruments (GE Healthcare, Uppsala, Sweden), and CM5 sensor chips, as described by Winquist et al. [31]. In brief, the NS5B Δ21 polymerase was immobilized by standard amine coupling to the level of 4000–5000 response units (RU). A buffer containing 10 mM 4-(2-hydroxyethyl)-1-piperazineethanesulfonic acid (HEPES) pH 7.5, 400 mM NaCl, and 3 mM dithiothreitol (DTT) was used during the immobilization procedure.

For determination of the direct interaction with NS5B, the thumb II inhibitors were diluted in the running buffer (20 mM Tris-HCl pH 7.4, 130 mM NaCl, 10 mM MgCl_2_, 0.05% (*v*/*v*) Tween 20) to a two-fold dilution series ranging from 1 to 0.0312 µM for experiments with NS5B Δ21 3a, and from 125 to 3.9 nM for NS5B Δ21 1b Con1 and BK. The inhibitors were injected in order of increasing concentration. The palm inhibitors were diluted in a running buffer containing 40 mM Tris-HCl (pH 7.5), 4 mM MgCl_2_, 4 mM DTT, 40 mM NaCl, and 10% glycerol, to a two-fold dilution series from 1 to 0.0625 µM. The DMSO concentration was kept at 3% (*v*/*v*) throughout the interaction experiments. The assay was performed at 25 °C with samples injected over the surface at a flow rate of 30 µL/min. The equilibrium dissociation constant (*K*_D_) was determined by steady-state analysis, and kinetic rate constants by global fit of a series of sensorgrams at different concentration, using Biacore T200 Evaluation software, V 3.0 (GE Healthcare, Uppsala, Sweden).

The palm compounds were prepared similarly as described above. Since a test injection of dasabuvir showed that it dissociated very slowly from the surface and regeneration was not possible, a single cycle kinetics experiment was performed. A heterogeneous binding model was fitted to the sensorgram. This model assumes that the inhibitor can interact with two forms of the enzyme. Thus, it is described as two independent 1:1 interactions, and the kinetic parameters, as well as the equilibrium dissociation constants (*K*_D_), are obtained separately for each binding reaction:

$$\begin{array}{l} E_{1}+I\overset{k_{a1}}{\underset{k_{d1}}{⇌}}E_{1}I\\ E_{2}+I\overset{k_{a2}}{\underset{k_{d2}}{⇌}}E_{2}I \end{array}$$

where *k*_a1_ and *k*_d1_ are the association and dissociation rate constants for the *E*_1_I complex, while *k*_a2_ and *k*_d2_ are the association and dissociation rate constants for the complex *E*_2_I. Thus, the estimated equilibrium dissociation constants for the *E*_1_I and *E*_2_I complexes are *K*_D1_ and *K*_D2_, respectively. The data were fitted by non-linear regression analysis using the following rate equations:

$${\left[E_{1}\right]}_{t=0}=B_{\max1}\;\;\;\;\;\;\;\;\;\;\;\;\;\;\;\;\;\;\;\;\;\;\;\;\;\;\;\;\;\;\;\;\;\frac{d\left[E_{1}\right]}{dt}=-\left(k_{a1}\times \left[I\right]\times \left[E_{1}\right]-k_{d1}\;\times \left[IE_{1}\right]\right)$$

$${\left[E_{2}\right]}_{t=0}=B_{\max2}\;\;\;\;\;\;\;\;\;\;\;\;\;\;\;\;\;\;\;\;\;\;\;\;\;\;\;\;\;\;\;\;\frac{d\left[E_{2}\right]}{dt}=-\left(k_{a2}\times \left[I\right]\times \left[E_{2}\right]-k_{d2}\;\times \left[IE_{2}\right]\right)$$

$${\left[IE_{1}\right]}_{t=0}=0\;\;\;\;\;\;\;\;\;\;\;\;\;\;\;\;\;\;\;\;\;\;\;\;\;\;\;\;\;\;\;\;\;\;\;\;\;\;\frac{d\left[IE_{1}\right]}{dt}=-\left(k_{a1}\times \left[I\right]\times \left[E_{1}\right]-k_{d1}\;\times \left[IE_{1}\right]\right)$$

$${\left[IE_{2}\right]}_{t=0}=0\;\;\;\;\;\;\;\;\;\;\;\;\;\;\;\;\;\;\;\;\;\;\;\;\;\;\;\;\;\;\;\;\;\;\;\;\;\frac{d\left[IE_{2}\right]}{dt}=-\left(k_{a2}\times \left[I\right]\times \left[E_{2}\right]-k_{d2}\;\times \left[IE_{2}\right]\right)$$

where B_max1_ and B_max2_ are the maximum binding capacities of *E*_1_ and *E*_2_, respectively.

### 2.5. Analysis of RNA–NS5B Interactions

Analysis of the direct interaction between RNA and NS5B was done using a Biacore S51 instrument (GE Healthcare) at 25 °C, essentially as described earlier [31]. The 5′ biotinylated RNA: 5′ CGAUACUCCCUUUAUAUAACCAUCAAUCGCC 3′ was purchased from Eurofins Genomics (Ebersberg, Germany). It was designed to contain two or more cytidine residues at the 3′ end and to have less probability to form a secondary structure, suitable for the de novo assay. Streptavidin (Sigma-Aldrich, St. Louis, MO, USA) was diluted to 250 µg/mL in a buffer containing 10 mM NaAc (pH 5), 0.1 mM ethylenediaminetetraacetic acid (EDTA), 1 mM NaCl, 1 mM DTT and immobilized to a CM5 chip surface by standard amine coupling procedures, resulting in an immobilization level of ~3000 response units (RU). Prior to immobilization of biotinylated RNA, the surface was washed three times with conditioning solution (1 M NaCl, 50 mM NaOH) to remove excess of unbound streptavidin. Biotinylated RNA was diluted in the buffer (10 mM Tris-HCl pH 8, 50 mM NaCl, 1 mM EDTA, 1 mM β-mercaptoethanol, and 0.005% Tween) and immobilized on the streptavidin surface resulting in ~700 RU. NS5B Δ21 1b and 3a were diluted in the running buffer (10 mM Tris-HCl, 150 mM NaCl, 10 mM MgCl_2_, 1 mM DTT, 5% glycerol) before injection over a chip surface with immobilized biotinylated RNA. A two-fold dilution series of the enzyme, ranging from 500 to 15.6 nM, was injected in order of increasing concentration. The palm inhibitors were mixed with the protein in a molar excess to an end concentration of 2 µM, while thumb inhibitors were mixed to 1 µM prior injection. Filibuvir and lomibuvir were found to have a greater effect on RNA binding to NS5B 1b; the test concentration of 1 µM was therefore sufficient. Smaller effects were seen for the thumb inhibitors to the NS5B 3a, and for the palm inhibitors for both NS5B 1b and 3a, motivating the use of a higher concentration (2 µM). The DMSO concentration in the protein running buffer was adjusted accordingly. After each cycle the surface was regenerated with 2 M NaCl and 2 M MgCl_2_ solution. All buffers were prepared in diethylpyrocarbonate (DEPC) (Sigma)-treated water. The data were evaluated with T100 Evaluation software, V.1.0 (GE Healthcare).

### 2.6. In Vitro RNA Synthesis

Two DNA oligos were used as a primer and a template, respectively, for 31-mer single-stranded RNA (ssRNA) synthesis in the polymerase assay on the biosensor chip: 5′ ATTCGTTAATACGACTCACTATAGGG 3′ and 5′ GGCGATTGATGGTTATATAAAGGGAGTATCGCCCTATAGTGAGTCGTATTA 3′. The primers were annealed by heating to 100 °C for 1 min in 50 mM Tris-HCl pH 7.4 and 100 mM KCl buffer. The RNA was synthesized using Riboprobe T7 kit (Promega, Fitchburg, WI, USA) according to manufacturer’s instructions. The synthesized RNA was purified with standard phenol/chloroform extraction procedure. Unincorporated nucleotides were removed using PD SpinTrap G-25 spin columns (GE Healthcare). The concentration was measured using a Nano-drop spectrophotometer (ND-1000, NanoDrop Technologies Inc., Wilmington, DE, USA).

### 2.7. Polymerase Activity Assay

**Figure 2:**
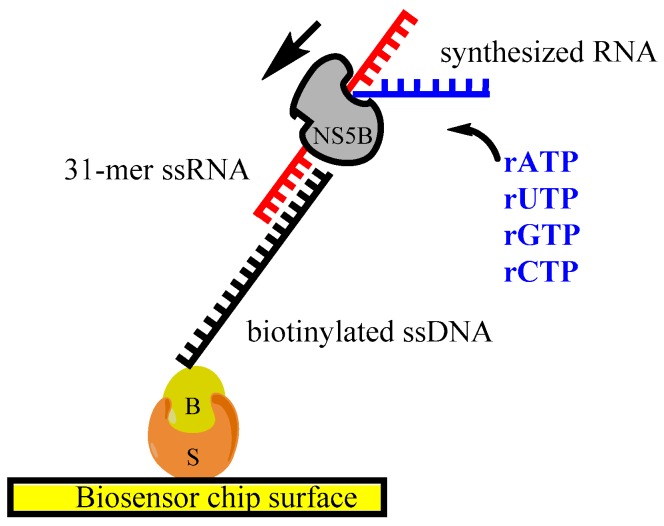
Illustration of the polymerase assay set-up on the biosensor chip using DNA/RNA hybrid template. The biotinylated single-stranded DNA (ssDNA) was immobilized on chip via streptavidin/biotin interaction. Subsequently, a 31-mer RNA was hybridized to the DNA by 8 complementary base pairs. The polymerase activity was monitored in real-time by injecting NS5B with nucleotides over the immobilized DNA/RNA hybrid. S = streptavidin, B = biotin.

The catalytic activity of HCV NS5B polymerase was estimated by Biacore X100 and T200 surface plasmon resonance biosensor instruments (GE Healthcare). The experimental setup is illustrated in Figure 2. Streptavidin (Sigma) was diluted to 100 µg/mL in the buffer containing 10 mM NaAc pH 5, 0.1 mM EDTA, 1 mM NaCl, 1 mM DTT and immobilized on CM5 chip surface by standard amine coupling procedure, resulting in 4500 RU. The surface was washed three times with conditioning solution (1 M NaCl, 50 mM NaOH) to remove excess of unbound streptavidin. Subsequently, about 500–700 RU of biotinylated ssDNA oligo diluted to 660 nM in the running buffer (10 mM Tris-HCl pH 7.4, 50 mM NaCl, 1 mM EDTA, 1 mM β-mercaptoethanol and 0.005% Tween 20) was captured by the streptavidin–biotin interaction. This ssDNA oligo has a complementary sequence to the in vitro synthesized single-stranded RNA, which is hybridized with an 8-base-pair overlap in the next step to an approximate level of 200–300 RU and subsequently becomes the template for an RNA synthesis by NS5B. The ssRNA oligo was diluted in the same buffer as ssDNA to a final concentration of 20 ng/µL. The NS5B Δ21 polymerase and/or NS5B Δ21 supplemented with 750 µM ribonucleotide triphosphates (rNTPs) (Promega) were injected over the surface containing DNA/RNA hybrid and over the reference surface with immobilized streptavidin (Figure 2).

The effect of inhibitors on polymerase activity was tested by adding 600 nM of filibuvir or lomibuvir to the NS5B/rNTPs mixture. The data was evaluated with BIAEvaluation v. 4.1 and T200 Evaluation software (GE Healthcare).

### 2.8. Sequence and Structure Analysis

A multiple sequence alignment of NS5B 1b (Con1), NS5B 1b (BK), and NS5B 3a was performed using web-based Clustal Omega [33]. The 3D structure of NS5B 3a was predicted using the online protein structure prediction server RaptorX [34]. The prediction used 4RY4 as a template structure [35].

## 3. Results

### 3.1. Stabilization of the Thermal Unfolding of NS5B by Allosteric Inhibitors

**Figure 3:**
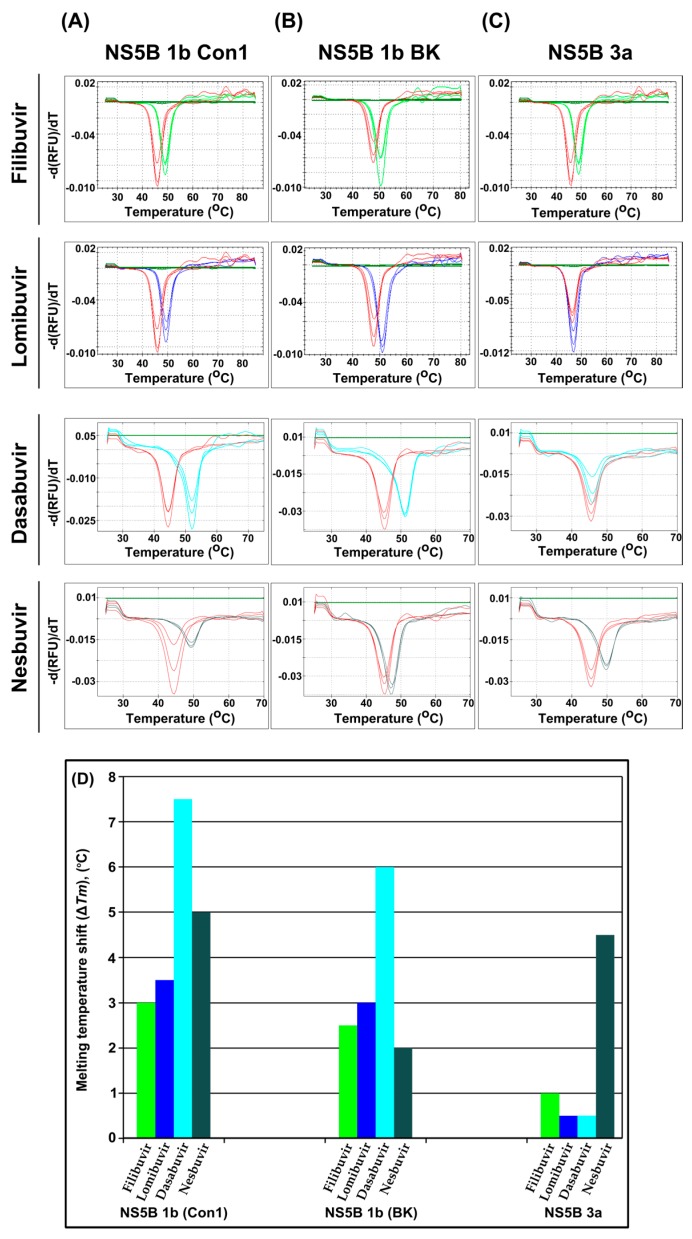
Effect on the thermal melting point of NS5B upon binding of thumb II inhibitors filibuvir (green), lomibuvir (blue) and palm inhibitors dasabuvir (turquoise), nesbuvir (grey). Thermal denaturation profile of NS5B 1b Con1 (**A**), NS5B 1b BK (**B**) and NS5B genotype 3a (**C**). Graphs representing the melting points of NS5B without inhibitors are in red. A summary of the thermal melting point shift (**Δ*T*_m_**) upon binding of the inhibitors to the tested NS5B variants is provided (**D**).

As a first comparison of the binding of allosteric inhibitors to genotypic variants of NS5B, the thermal stability of the protein was evaluated in the absence and presence of inhibitors by differential scanning fluorimetry (Figure 3). The thermal melting points (*T*_m_) of NS5B variants were similar, although they varied slightly for different protein batches (Table 1). The experiments demonstrated that thumb II inhibitors only induced a minor shift in the *T*_m_ for genotype 3a NS5B, i.e., 1 °C for filibuvir and 0.5 °C for lomibuvir (Figure 3C). Although there was little effect by one of the palm inhibitors, dasabuvir (0.5 °C), the other, nesbuvir, shifted the *T*_m_ of NS5B 3a by 4.5 °C (Figure 3C). In contrast, all allosteric inhibitors affected the *T*_m_ of NS5B from genotype 1b strain Con1 significantly (Figure 3A). It was shifted 3 °C to 3.5 °C by filibuvir, lomibuvir and nesbuvir and 7.5 °C with dasabuvir. The presence of dasabuvir had an almost similar effect on NS5B 1b isolate BK and shifted the *T*_m_ by 6 °C, whereas nesbuvir only shifted the *T*_m_ by 2 °C, indicating that the interaction is weak. The data suggest that nesbuvir is the only allosteric inhibitor that interacts significantly with the polymerase from genotype 3a.

### 3.2. Kinetic Analysis of Interactions between Inhibitors and NS5B

**Figure 4:**
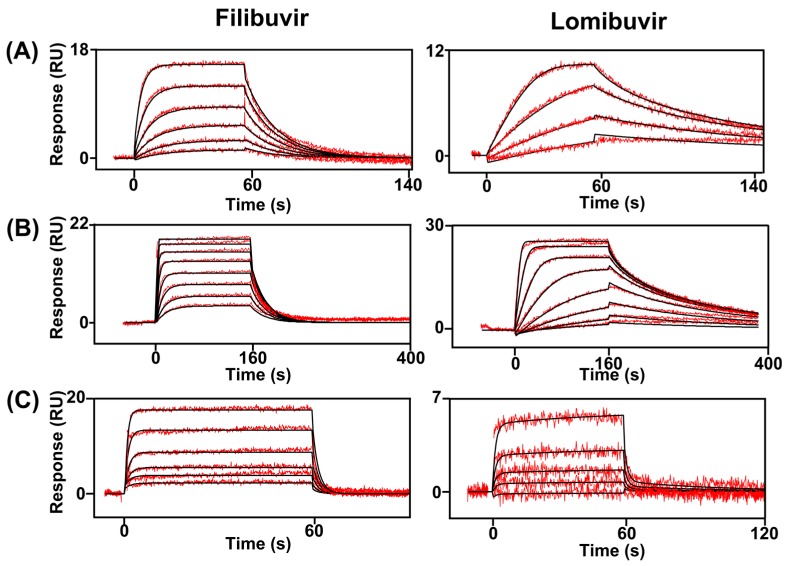
Interaction of thumb II inhibitors and sensor surfaces with immobilized NS5B from different genotypes. A two-fold dilution series, ranging from 1 to 0.0312 µM, was injected in order of increasing concentration. (**A**) Genotype 1b, isolate Con1; (**B**) genotype 1b, isolate BK; and (**C**) genotype 3a. The sensorgrams are blank and reference surface subtracted. The black lines correspond to fitted interaction models. RU: response units.

The interactions between the allosteric inhibitors and NS5B were further analyzed using an SPR biosensor assay since this assay has a greater sensitivity and is more informative than the DSF assay (Figure 4). It demonstrated that filibuvir and lomibuvir both interact with NS5B 3a, although the thermal shift assay did not show strong evidence of binding (Figure 4C). To quantify the differences in kinetics and estimate the rate constants, the data were fitted to a 1:1 Langmuir interaction model or a two-state model (induced fit). The simpler model was adequate for the filibuvir interaction, but the interactions with lomibuvir were best described by a two-state reaction model for both genotypes (Table 2). The affinity of filibuvir to NS5B 3a was 10 times lower than for NS5B 1b, and lomibuvir had a significantly lower affinity for NS5B 3a than 1b. The reduced affinities were due to both slower association rates and faster dissociation rates than those with NS5B 1b (Table 2). There were no major differences in the interaction profiles of studied compounds between isolates Con1 and BK of genotype 1 the affinities and rate constants for the two genotypes were similar.

**Figure 5:**
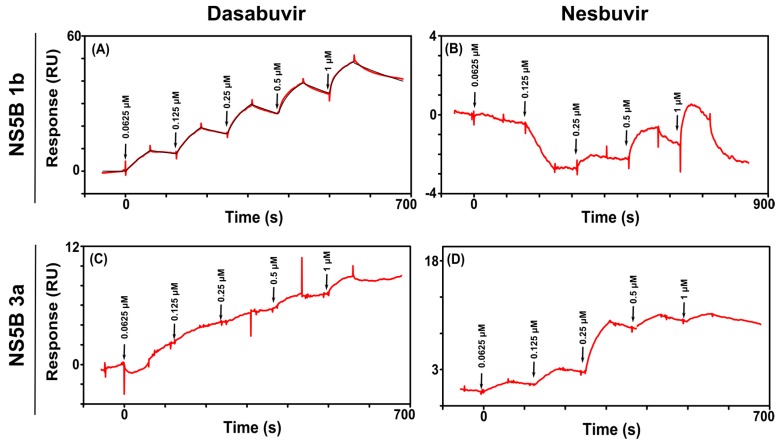
Interaction of dasabuvir with an NS5B 1b Con1 sensor surface (**A**). A two-fold dilution series, ranging from 1 to 0.0625 µM, was injected in order of increasing concentration. The single-cycle kinetic sensorgram is the reference subtracted. The black line corresponds to the heterogeneous model fitting. The calculated interaction parameters are presented in Table 3. Dilution series injections of nesbuvir over immobilized NS5B 1b Con1 (**B**). Injections of dasabuvir (**C**) and nesbuvir (**D**) over immobilized NS5B 3a.

Dasabuvir showed a strong binding to NS5B 1b, with a very slow dissociation rate. As the surface could not be regenerated, a single cycle kinetic experiment was performed in order to quantify the kinetics (Figure 5A, Table 3). The interaction was complex and best described by a heterogeneous model. Dasabuvir was not seen to interact with NS5B 3a (Figure 5C).

In contrast to what was expected on the basis of the thermal shift analysis, no interaction was detected between nesbuvir and NS5B 1b (Figure 5B). The binding of nesbuvir to NS5B 3a was observed, and also exhibited a slow dissociation rate. The interaction could not be described by any pre-defined models (Figure 5D).

### 3.3. Effects of Allosteric Inhibitors on Interactions between NS5B and RNA

**Figure 6:**
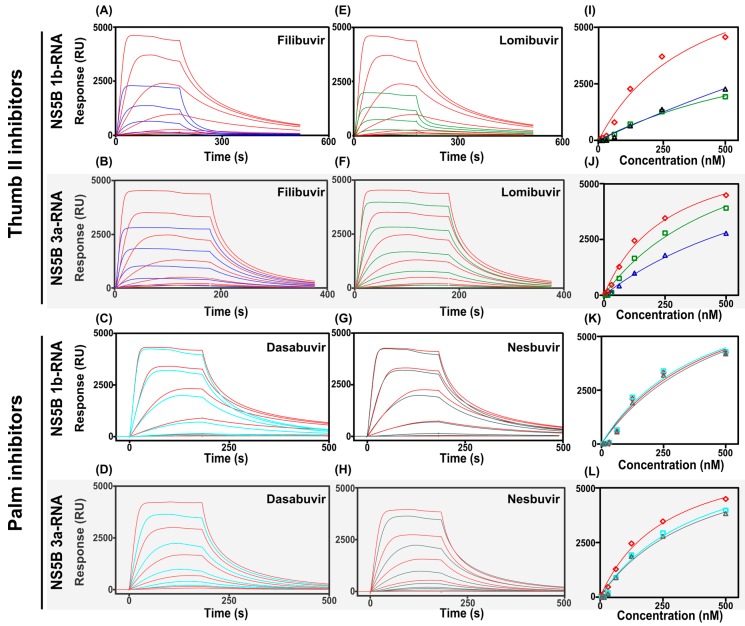
The effect of inhibitors on the interaction between NS5B from genotype 1b or 3a injected over the surface with immobilized RNA. NS5B 1b were injected in two-fold serial concentrations between 15.6 and 500 nM without inhibitors (red sensorgrams) and in the presence of filibuvir (**A**), lomibuvir (**E**), dasabuvir (**C**) or nesbuvir (**G**). The concentrations of thumb and palm inhibitors kept constant at 1 µM (**A**,**E**) or 2 µM (**B**–**D**,**F**–**H**), respectively. NS5B 3a were injected in two-fold serial concentrations between 15.6 and 500 nM without inhibitors (red sensorgrams) and in the presence of filibuvir (**B**), lomibuvir (**F**), dasabuvir (**D**) or nesbuvir (**H**). (**I**–**L**) show the concentration-dependent signals at 75 s of NS5B 1b and 3a injected alone (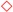) and in complex with filibuvir (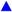), lomibuvir (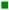), dasabuvir (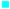), and nesbuvir (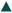). The corresponding equilibrium dissociation constants (*K*_D_) determined by a 1:1 steady-state affinity model are presented in Table 4.

In order to elucidate the mode-of-action of the allosteric thumb II and palm pocket inhibitors, the possible interference on the interaction between RNA and NS5B 1b Con1 and 3a was investigated using an SPR biosensor-based assay. The experiment estimated the binding of NS5B, injected as an analyte, to biotinylated RNA, captured to the sensor surface via immobilized streptavidin. The interaction was apparently saturable and allowed steady-state analysis for determination of the approximate affinity of NS5B for RNA (Figure 6). Both NS5B variants bound to RNA and had comparable nanomolar affinities (Table 4). The data were fitted assuming a simple one site binding model, but the interaction appears to be more complex. The affinity values are therefore approximate estimations.

The binding of both genotypic variants of NS5B to RNA was reduced when the protein was injected together with the thumb compounds filibuvir or lomibuvir (Figure 6A,B,E,F). The effect was both on the signal level (Figure 6) and on the affinities (Table 4). Lomibuvir has the largest effect on the NS5B 1b interaction, reducing the NS5B–RNA affinity significantly (Figure 6E). Filibuvir displayed the largest effect on the NS5B 3a–RNA interaction, decreasing the binding affinity about three-fold (Figure 6B).

The palm compounds were not seen to interfere with the binding of NS5B 1b and 3a to RNA (Figure 6C,D,G,H, Table 4).

### 3.4. Real-Time Monitoring of the RNA Polymerization by HCV NS5B

**Figure 7:**
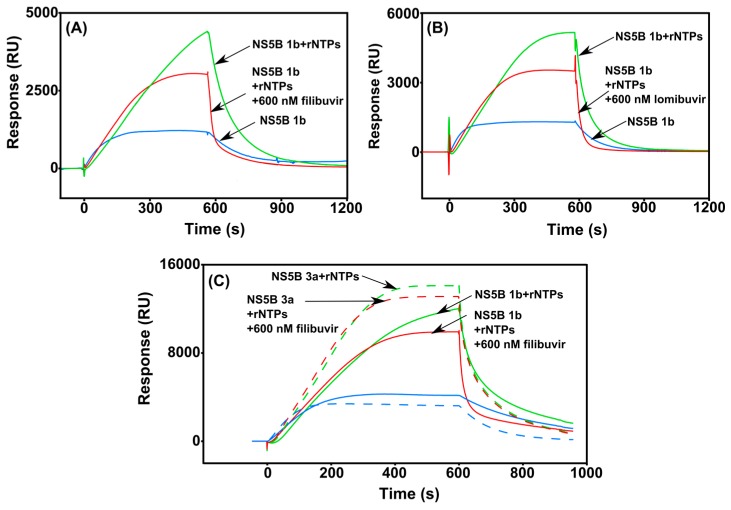
On-chip nucleotide incorporation into RNA by the HCV NS5B polymerase. The effect of the inhibitors filibuvir (**A**) and lomibuvir (**B**) on the incorporation of nucleotides by NS5B 1b (red line), compared to without inhibitors (green) was monitored. (**C**) Comparison of the incorporation of nucleotides by NS5B (green) and the effect of filibuvir present (red) between genotype 1b Con1 (solid lines) and 3a (dashed lines). Controls NS5B alone without added ribonucleotide triphosphates (rNTPs) and without inhibitor are shown in blue. The samples contained 100 nM NS5B, ±750 µM rNTPs and ±600 nM inhibitor.

In order to assess the activity of the in-house produced NS5B polymerases and to understand the mode-of-action of allosteric compounds, we developed a biosensor-based polymerase assay. The elongation of a synthesized RNA chain by NS5B was monitored in real time on a biosensor chip (Figure 2). The observed polymerase activity was detected as a continuous increase of the signal, as shown in Figure 7. The sensorgrams show that the signals are higher and continue to rise during the 9 min injection of samples containing both NS5B and rNTPs. A slow dissociation was observed after the injection stop, representing the termination of elongation and release of the enzyme from the template. The signal returned back to baseline after dissociation, indicating that the newly synthesized RNA dissociated from the immobilized template together with the enzyme. Controls with only NS5B and no rNTPs also gave a signal increase, interpreted as a result of NS5B binding to the template. To confirm that the signals were not dependent on the assay set-up, the sample injection order was randomized. However, this did not influence the response profile (not shown).

When the enzyme was injected together with rNTPs and inhibitor, the response levels were significantly lower, demonstrating inhibition of binding of the enzyme to the template RNA. The signals were higher than the controls without rNTPs, indicating that inhibition was only partial under these conditions (Figure 7). It is notable that the injection of NS5B polymerase together with either filibuvir or lomibuvir showed rapid dissociation (Figure 7A,B). These results support the experiments above and that the thumb inhibitors interfere with the interaction between RNA and NS5B. As expected, from the DSF and biosensor assays, thumb II inhibitors had no effect on the polymerase activity of NS5B 3a compared to NS5B 1b (Figure 7C).

Considering that the incorporation of one nucleotide will theoretically result in a signal of about 4.6 RU [36], the nucleotide incorporation rate was calculated to be about 290 bases per minute for NS5B 1b. NS5B 3a exhibited a similar activity, with a nucleotide incorporation rate of about 350 bases per minute (Figure 7C). Neither of the thumb II compounds had an effect on the rNTPs incorporation rate.

### 3.5. Sequence Comparison and Structure Analysis

**Figure 8:**
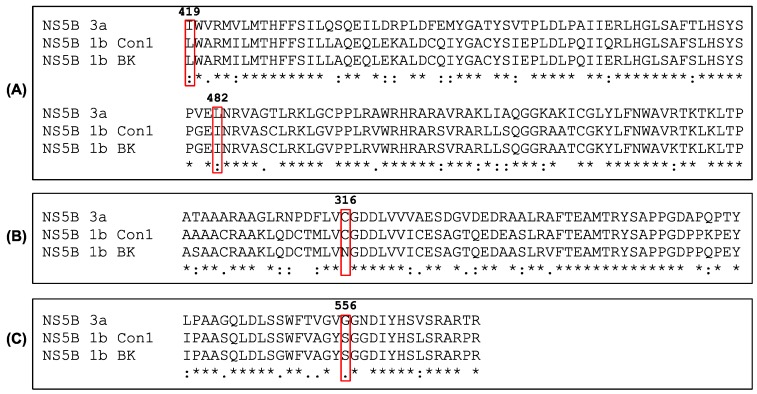
Alignment of sequences for the allosteric sites of NS5B from genotypes 1b with the corresponding sites in NS5B 3a. (**A**) filibuvir site, (**B**) nesbuvir site and (**C**) dasabuvir site. Residues previously identified to interact with filibuvir and confer resistance to all known thumb II allosteric inhibitors are boxed.

A sequence and 3D structure analysis of the studied NS5B variants was performed in order to understand the possible cause of the low affinity of the allosteric inhibitors for the genotype 3a polymerase. Overall, NS5B genotype 1b and genotype 3a sequences alignment showed about 77% of sequence identity and 96.5% between NS5B 1b Con1 and BK isolates. We focused on the residues in the binding pockets for studied allosteric inhibitors in NS5B 1b. The sequence analysis (Figure 8) shows that two amino acids in the filibuvir binding site of NS5B are different in NS5B 3a, i.e., L419I and I482L. Substitutions in both positions are known to be associated with genotype 1b resistance towards various non-nucleoside ligands binding in thumb pocket II [29]. Examination of palm site sequences revealed resistance-associated substitutions at S556G in the NS5B 3a sequence as compared to both 1b variants, and C316N in the 1b BK sequence [13,37].

**Figure 9:**
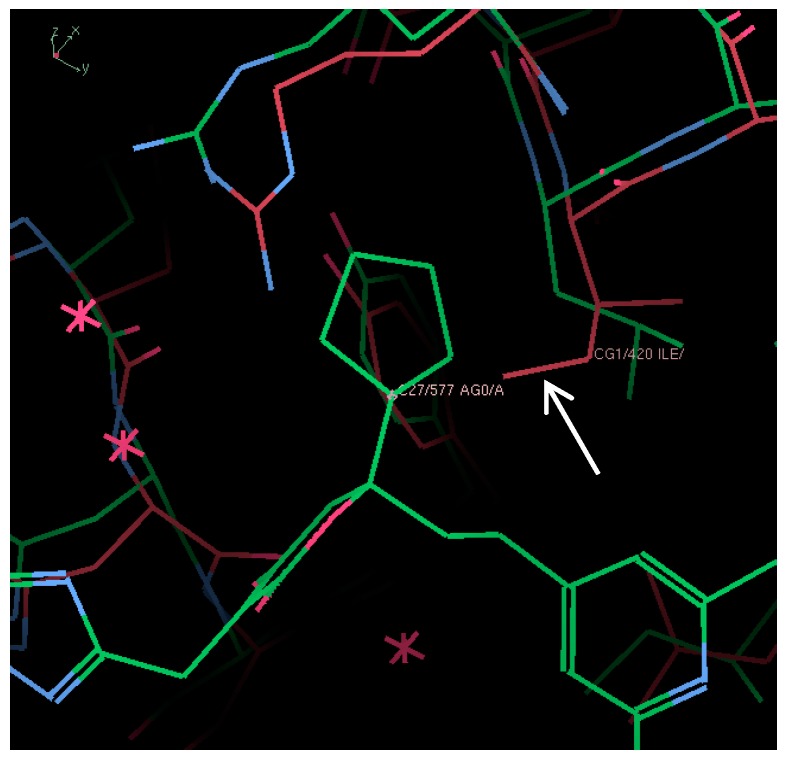
Close up view of thumb II binding site of NS5B 1b with filibuvir (PDB ID: 3FRZ) (green) superimposed with predicted structure of NS5B 3a (red). Residue L419 partially protrudes into the binding site of the cyclopentyl group of the inhibitor (white arrow).

The positions of the identified residues were compared in NS5B from genotype 1b and 3a by first predicting the 3D structure of NS5B 3a since a crystal structure of NS5B 3a polymerase could not be obtained. The structure of NS5B 1b (BK) was superimposed in complex with filibuvir (PDB ID: 3FRZ). The predicted NS5B 3a structure did not superimpose perfectly with the 1b structure, probably a consequence of using the genotype 1b strain J4 structure as a template for the prediction instead of the BK strain structure. Nevertheless, examination of the thumb II site of aligned predicted NS5B 3a and NS5B 1b with filibuvir indicates that the substitution at the position L419 is likely to be a major cause of impaired filibuvir binding due to a steric hindrance of its cyclopentyl group with isoleucine (Figure 9).

## 4. Discussion

In this study, we have characterized the interaction between two classes of allosteric non-nucleoside HCV NS5B inhibitors with their target. By using a combination of direct binding assays, and indirect assays that monitor effects on the interaction between the enzyme and RNA, and on the catalytic activity of the enzyme, we could show that the two classes of compounds had different modes-of-action and that they displayed a large genotypic variation in inhibitor interactions. The biophysical analyses were supported by sequence and structure analyses, which identified residues that differ in the allosteric binding sites of genotypes 1b and 3a.

### 4.1. Binding

An initial analysis involved a comparison of the ability of the allosteric inhibitors to bind to their proposed targets, a fundamental feature of any inhibitor, but less trivial for allosteric inhibitors due to their indirect mode-of-action. Here we were also interested in identifying differences between the enzyme variants from different genotypes.

The two studied thumb inhibitors interacted with all three NS5B variants, although the affinities for the 3a enzyme were low due to fast dissociation kinetics and gave a smaller *T*_m_ shift for NS5B 3a compared to NS5B 1b. Although filibuvir and lomibuvir have been deduced to have the same or partially overlapping binding site, they may be influenced to different degrees by the genotype variations. For example, hydrophobic residues in thumb pocket II, including L419 and I482, differ between genotype 1b and 3a [26,38,39]. These appear to contribute to the interaction with a hydrophobic part of lomibuvir, and may similarly also be involved in lomibuvir resistance [29,31,40]. Although single mutations of NS5B 1b, such as I482L and L419M, have a modest effect on the binding of filibuvir, according to SPR and replicon assays, they increased the 50% effective concentration (EC_50_) of lomibuvir, 100-fold and 9-fold, respectively [29]. It is consequently likely that the structure of the putative NS5B 3a thumb pocket II is not suited for binding of the compounds, apparently due to a narrower binding site.

The interaction kinetics and DSF data of thumb inhibitors with NS5B 1b (Con1) variant presented here are in an agreement with our previous study [31] and with other studies. However there are some small variations in DSF data, which can be explained by different experimental set ups, and SPR data especially with lomibuvir, where the interactions were described by a simple 1:1 interaction model [29,40,41].

Of the two tested palm inhibitors, only dasabuvir interacted with the 1b variant with high affinity, and neither interacted with NS5B 3a. Dasabuvir also stabilized the structure of the two 1b variants, but not the 3a enzyme. The SPR interaction between dasabuvir and NS5B 1b was best described by a heterogeneous model, presumably as a result of covalent attachment of NS5B polymerase on the biochip surface causing appearance of two forms of the enzyme. As expected, no interaction was detected between NS5B 3a and dasabuvir, most likely due to the presence of the mutation S556G, which was shown to confer the resistance to dasabuvir [13]. The resistance to nesbuvir has not been reported for NS5B 3a [42]; the interaction between NS5B 3a and nesbuvir was observed in the SPR assay but not with NS5B 1b (Con 1). Also, the DSF experiment demonstrated a distinct shift in the melting point of NS5B 3a in presence of nesbuvir. This strongly suggests a direct interaction between nesbuvir and NS5B 3a. Its slow dissociation was also seen in a previous study with 1b (Con1) enzyme variant using BioRad ProteOn SPR instrument and the interaction could not be described by a simple 1:1 interaction model [41]. The lack of interaction of NS5B 1b (Con1) with the nesbuvir and poor quality sensorgram obtained with NS5B 3a in the SPR assay may be explained by structural rearrangements of NS5B when immobilizing NS5B to the SPR sensor chip leading to full or partial inaccessibility of the palm binding site by reducing the structural flexibility of the enzyme.

### 4.2. Effect on RNA-Binding Only or Catalytic Mechanism?

For the inhibitors that were confirmed to bind to the target, it was of interest to elucidate how this translates into inhibition. The effect of an allosteric inhibitor on enzyme functionality can be significant, even when binding only results in small conformational changes. Such minor changes may be sufficient to alter, for example, the electrostatic properties of the active site or cause a “population shift” of structurally dynamic proteins such as NS5B polymerase [43]. Here we were interested in deciphering if the induced effect was simply from preventing the enzyme from binding its substrate, in the form of RNA, or if it interfered with the catalytic mechanism via other structural effects.

#### 4.2.1. Filibuvir and Lomibuvir

Our experiments demonstrated that the interaction between RNA and NS5B was reduced in the presence of thumb inhibitors. This is in accordance with previous data that has indicated that thumb pocket inhibitors induce a conformational change upon binding and that the inhibitor has a capacity to reduce the space between the thumb and the fingers domains although thumb pocket II is located approximately 35 Å from the active site [29,39]. This consequently appears to induce a conformational state that may be less capable of binding RNA to the active site [44]. However, this may not be the complete effect of inhibitor binding as it has been reported that the presence of a thumb inhibitor reduces the overall flexibility and the movement between open and closed state of the enzyme, essential for catalytic activity [25,44,45].

#### 4.2.2. Dasabuvir and Nesbuvir

In contrast to the thumb domain inhibitors, the palm domain inhibitors were here found to have little or no effect on the interaction between NS5B and RNA, suggesting a different mode of action of this class of inhibitors. The palm inhibitor binding pocket is located closer to the active site than the binding pocket for the thumb II inhibitors and binding of a ligand to this site can consequently have a different effect to that induced by thumb site inhibitors [45]. The inhibitory effect of nesbuvir has been suggested to be associated with nucleotide incorporation interference and/or impairment of ssRNA and dsRNA recognition [46], thus blocking the initiation stage of the polymerization reaction. A similar mechanism was suggested for dasabuvir [12]. Our results, showing no influence on the binding of NS5B to RNA in the presence of the palm site inhibitors, support such a mode of inhibition.

### 4.3. Inhibition

#### 4.3.1. Assay Development

In order to quantify the relationship between binding and inhibition, we established an SPR-based de novo polymerase assay, which allowed the estimation of the rate of rNTP incorporation into RNA by NS5B. Despite the differences in the NS5B sequence between genotypes 1b and 3a, we found the catalytic activities to be almost similar, however, NS5B3a showed a slightly higher rNTP incorporation rate.

The accuracy of our established assay was demonstrated by the observed nucleotide incorporation rate of NS5B 1b of 290 nucleotides per minute, which is close to the previously reported incorporation rate of 150–200 bases per minute estimated by scintillation count assay [47]. As previously shown [48], a high concentration of rNTPs was required for de novo RNA synthesis by NS5B. Below 500 µM of rNTPs concentration the de novo activity was reduced significantly (not shown). Therefore, the estimation of inhibitory effect of nucleoside analogues is challenging using the de novo assay set-up presented herein.

A previous study revealed that one product of de novo synthesis by NS5B polymerase is single-stranded RNA [49]. We observed that after dissociation, the baseline almost returned to the same level as before injection start. This is an indication of ssRNA synthesis and its release together with NS5B, if dsRNA had been formed, it would have resulted in the accumulation of product on the surface and an increased baseline level. Next, the design of the RNA template used in the assay prevented formation of self-priming hairpin ensuring de novo synthesis including 3 cytidylates in a row at the 3′ end of the RNA template [50]. This was important, although recombinant NS5B polymerase is able to copy the entire HCV RNA in vitro, when it somehow is able to unwind secondary and tertiary RNA structures [51,52,53,54].

#### 4.3.2. Application of the Assay for Inhibition Studies

The catalytic assay demonstrated that both filibuvir and lomibuvir were potent inhibitors of NS5B 1b Con1 polymerase. However, there was no significant inhibition of NS5B 3a by either of the compounds in the conventional radioactive nucleotide incorporation (data not shown) or in the SPR-based polymerase assay with filibuvir. This is in accordance with a previous study that reported that the half maximal inhibitory concentration (IC_50_) value of filibuvir for NS5B 3a is about 70-fold greater than against NS5B 1b Con1 [55].

We observed that neither filibuvir nor lomibuvir reduced the response level to or below the binding level of NS5B to RNA. The activity was still observed in presence of inhibitors, however, the curves reached a plateau earlier than without inhibitors. This could indicate that thumb pocket II inhibitors may interfere with the initiation process [56]. Moreover, a fast dissociation from the template RNA was observed in presence of both thumb II inhibitors, confirming their interference with the NS5B–RNA complex.

The palm compounds did not inhibit the polymerase activity of either NS5B variants using this assay set-up (not shown). A longer incubation time is probably required, due to a slow binding mechanisms of palm inhibitors, as previously reported for nesbuvir [27].

## 5. Conclusions

In this study, we illustrate the advantage of biophysical methods in evaluating the mode-of-action of HCV NS5B polymerase inhibitors and to compare their efficacies against different HCV genotypes. This is important as these differences are likely to directly reflect the in vivo efficacy. Furthermore, we demonstrated that SPR technology can be used for continuous monitoring of the HCV NS5B enzymatic activity and its inhibition in real time, thus providing a valuable tool for further drug discovery of polymerase inhibitors.

Previous studies have demonstrated that thumb II inhibitors lock the closed, inactive conformation of the enzyme, while not being able to bind to the open, active conformation [41,57], which occurs during the elongation phase [58]. In addition, the closed conformation reduces the binding of the enzyme to ssRNA [59]. However, closed conformation is required for the initiation step of the RNA synthesis [58]. Our observations are in an agreement with a recent study that has reported the accumulation of abortive 4 and 5 nucleotide intermediates in the presence of thumb II non-nucleoside inhibitors, and they do not have any significant effect neither on the initiation rate nor on elongation rate [60]. Consequently, it is most likely that thumb II inhibitors trap the closed conformational state of the NS5B, reducing the flexibility, which is required for the activity, and therefore block the progression from closed to open conformation and thus the transition from initiation to elongation.

The information presented in this study is useful to discover and develop new potent allosteric DAAs against HCV NS5B polymerase from genotype 3a as well as to get insights of resistance profiles that may arise during HCV treatment with these compounds.

## Figures and Tables

**Table 1:** Effect of allosteric hepatitis C virus (HCV) NS5B inhibitors on the thermal stability of NS5B from genotypes 1b (strains Con1 and BK) and 3a.

Inhibitor	NS5B	1b Con1		1b BK		3a	
	(°C)	*T*_m_	Δ*T*_m_	*T*_m_	Δ*T*_m_	*T*_m_	Δ*T*_m_
- ^a^		46.0 (44.5) ^b^		48.0 (45.0) ^b^		46.5 (45.5) ^b^	
Filibuvir		49.0	3	50.5	2.5	47.5	1
Lomibuvir		49.5	3.5	51.0	3	47.0	0.5
Dasabuvir		52.0	7.5	51.0	6	46.0	0.5
Nesbuvir		49.5	5	47.0	2	50.0	4.5

^a^ Control with dimethyl sulfoxide (DMSO) in buffer at the same concentration as in the inhibitor sample. ^b^ Values estimated when measurement of palm inhibitors was performed, i.e., with a new batch of produced proteins.

**Table 2:** Kinetic parameters for filibuvir and lomibuvir interacting with NS5B from genotype 1b (isolates Con1 and BK). The parameters were determined using a 1:1 Langmuir model for filibuvir and a two-step model for lomibuvir.

	Filibuvir			Lomibuvir		
	1b Con1 (±SD) ^d^	1b BK (±SD)	3a (±SD)	1b Con1 (±SD)	1b BK (±SD)	3a (±SD)
*K*_D_ (nM) ^b^	55 ± 11 (30 ± 6)	54 ± 1.4 (30 ± 11)	545 ± 7 (800 ± 80)	Nd **^a^** (19 ± 7)	Nd **^a^** (20 ± 0.4)	>2000 (2600)
*k*_a_ (M^−1^s^−1^)	2.6 × 10^6^ ± 1.1 × 10^6^	1.84 × 10^6^ ± 0.22 × 10^6^	7.0 × 10^5^ ± 0.2 × 10^5^	2.1 × 10^6^ ± 1.1 × 10^6^	1.8 × 10^6^ ± 0.22 × 10^6^	1.75 × 10^5^ ± 1.05 × 10^5^
*k*_d_ (s^−1^)	0.07 ± 0.02	0.03 ± 0.008	0.57 ± 0.06	0.04 ± 0.03	0.03 ± 0.007	0.45 ± 0.21
*k*_2_ (s^−1^)	N/A ^c^	N/A	N/A	7.7 × 10^−6^ ± 3.1 × 10^−6^	2.6 × 10^−6^ ± 0.9 × 10^−6^	0.048 ± 0.06
*k*_-2_ (s^−1^)	N/A	N/A	N/A	0.001 ± 7 × 10^−5^	0.002 ± 0.0007	0.025 ± 0.007
*χ^2^*	0.49 ± 0.19	0.5 ± 0.2	0.16 ± 0.13	0.22 ± 0.2	0.66 ± 0.33	0.09 ± 0.02

^a^ Nd: Not determined. ^b^ Values estimated by steady-state analysis, values calculated from kinetic data are in parentheses. ^c^ N/A: not applicable. ^d^ The sample standard deviations (SD) for the averages are based on two experiments. K_D_: equilibrium dissociation constant.

**Table 3:** Interaction kinetic parameters for dasabuvir and NS5B from genotype 1b (isolate Con1). The parameters were determined using a heterogeneous model.

	Dasabuvir
	NS5B 1b Con1 (±SD) ^a^
*k*_a1_ (M^−1^ s^−1^)	1.5 × 10^4^ ± 0.3 × 10^4^
*k*_d1_ (s^−1^)	3.3 × 10^−7^ ± 0.2 × 10^−7^
*K*_D1_ (M)	2.3 × 10^−11^ ± 0.6 × 10^−11^
*k*_a2_ (M^−1^ s^−1^)	8.6 × 10^5^ ± 1.1 × 10^6^
*k*_d2_ (s^−1^)	5.1 × 10^−3^ ± 2.6 × 10^−3^
*K*_D2_ (M)	3.6 × 10^−8^ ± 0.9 × 10^−8^
*χ^2^*	0.4 ± 0.4

^a^ The sample standard deviations for the averages are based on two experiments.

**Table 4:** Effect of allosteric inhibitors on the interaction between NS5B from genotype 1b and 3a and RNA. The dissociation constants (*K*_D_) were estimated from sensorgrams shown in Figure 9, by steady-state affinity analysis.

	NS5 1b *K*_D_ (nM) ± SE	B_max_ (RU)	NS5B 3a *K*_D_ (nM) ± SE	B_max_ (RU)
NS5B	390 ± 149	8473	266 ± 49	7003
NS5B + Filibuvir	950 ± 446	5693	947 ± 319	8158
NS5B + Lomibuvir	3350 ± 4346	17,715	553 ± 185	8418
NS5B + Dasabuvir	430 ± 200	8340	407 ± 127	7364
NS5B + Nesbuvir	530 ± 243	8957	411 ± 134	7107

B_max_: maximum binding capacity calculated from the curve fit; SE: standard error obtained from the curve fit.
